# Docosahexaenoic Acid Ameliorates Fructose-Induced Hepatic Steatosis Involving ER Stress Response in Primary Mouse Hepatocytes

**DOI:** 10.3390/nu8010055

**Published:** 2016-01-20

**Authors:** Jinying Zheng, Chuan Peng, Yanbiao Ai, Heng Wang, Xiaoqiu Xiao, Jibin Li

**Affiliations:** 1 School of Public Health and Management, Chongqing Medical University, Research Center for Medicine and Social Development, Innovation Center for Social Risk Governance in Health, Chongqing 400016, China; jinyingzheng1988@163.com (J.Z.); aiyanbiao1992@163.com (Y.A.); 2 Laboratory of Lipid & Glucose Metabolism, The First Affiliated Hospital of Chongqing Medical University, Chongqing 400016, China; 13527441813@163.com (C.P.); hengyy663@163.com (H.W.)

**Keywords:** docosahexaenoic acid, fructose, ER stress, NAFLD

## Abstract

The increase in fructose consumption is considered to be a risk factor for developing nonalcoholic fatty liver disease (NAFLD). We investigated the effects of docosahexaenoic acid (DHA) on hepatic lipid metabolism in fructose-treated primary mouse hepatocytes, and the changes of Endoplasmic reticulum (ER) stress pathways in response to DHA treatment. The hepatocytes were treated with fructose, DHA, fructose plus DHA, tunicamycin (TM) or fructose plus 4-phenylbutyric acid (PBA) for 24 h. Intracellular triglyceride (TG) accumulation was assessed by Oil Red O staining. The mRNA expression levels and protein levels related to lipid metabolism and ER stress response were determined by real-time PCR and Western blot. Fructose treatment led to obvious TG accumulation in primary hepatocytes through increasing expression of fatty acid synthase (FAS) and acetyl-CoA carboxylase (ACC), two key enzymes in hepatic *de novo* lipogenesis. DHA ameliorates fructose-induced TG accumulation by upregulating the expression of carnitine palmitoyltransferase 1A (CPT-1α) and acyl-CoA oxidase 1 (ACOX1). DHA treatment or pretreatment with the ER stress inhibitor PBA significantly decreased TG accumulation and reduced the expression of glucose-regulated protein 78 (GRP78), total inositol-requiring kinase 1 (IRE1α) and p-IRE1α. The present results suggest that DHA protects against high fructose-induced hepatocellular lipid accumulation. The current findings also suggest that alleviating the ER stress response seems to play a role in the prevention of fructose-induced hepatic steatosis by DHA.

## 1. Introduction

Nonalcoholic fatty liver disease (NAFLD) has become the most common liver disease globally. It is estimated that 24% to 42% of the population in Western countries and 5% to 42% in Asian countries are affected [1,2]. NAFLD, a hepatic manifestation of metabolic syndrome, is characterized by an increase in intrahepatic triglyceride (*i.e.*, steatosis) in the absence of excessive alcohol intake. It can progress to nonalcoholic steatohepatitis (NASH) when hepatocellular injury and inflammation are present, and may lead to liver fibrosis and cirrhosis [3,4]. It is frequently associated with obesity and dyslipidemia, type 2 diabetes, insulin resistance and some dietary factors, such as high energy, fat and excess sugar intakes [5,6,7].

The consumption of sweetened foods and beverages, which contain high concentrations of fructose, has increased in the last few decades [8,9]. Increasing evidence indicates that high fructose intakes might be an important risk factor in the development of NAFLD [10,11,12]. Studies in both animals and humans have shown that high fructose consumption was associated with increased *de novo* lipogenesis, triglycerides synthesis and secretion of very low density lipoproteins, and decreased fatty acid oxidation and impaired insulin signaling [13,14,15,16,17].

Docosahexaenoic acid (22:6 *n*-3, DHA) and eicosapentaenoic acid (EPA), the major polyunsaturated fatty acids (PUFA) of *n*-3 series found in marine fish oil, are essential for mammals because they cannot be produced in the body and must be obtained from food. Some studies in humans and rodents demonstrated that dietary PUFA influenced hepatic triglyceride levels, insulin resistance and inflammation [18,19,20]. The beneficial effects of EPA and DHA supplementation on lipogenesis, fatty acid oxidation and hepatic lipid metabolism have been reported in numerous studies [20,21,22]. Some authors have recently demonstrated that the supplementation of *n*-3 fatty acids had potential therapeutic effects in human NAFLD as well as other metabolic disorders, such as insulin resistance, dyslipidemia, and impaired cognitive functions [11,23,24,25]. In addition, DHA and EPA can alter metabolic pathways, improve insulin sensitivity by modulating related gene expression and ameliorate hepatic triglycerides accumulation in rats fed a high-fructose diet [20]. These findings suggest that dietary supplements of PUFA may be beneficial for the patients with NAFLD. Nevertheless, the molecular mechanism that PUFA ameliorates NAFLD is not entirely clear.

Endoplasmic reticulum (ER) stress has long been proposed to play a crucial role in the development of NAFLD [26,27]. Interestingly, recent studies showed that the activation of ER stress pathways in high fructose-fed mice mediated *de novo* lipogenesis and then altered hepatic steatosis and insulin resistance [28]. It has been demonstrated that supplementation of *n*-3 fatty acids attenuated hepatic steatosis [11,23]. However, it remains unclear whether DHA prevents fructose-induce NAFLD by regulating ER stress pathways. In this study, we investigated the effects of DHA on hepatic lipid metabolism in fructose-treated primary mouse hepatocytes, and the changes of ER stress pathways in response to DHA treatment.

## 2. Materials and Methods

### 2.1. Materials and Reagents

DHA (purity ≥ 98%), Oil Red O, tunicamycin, insulin, dexamethasone, rat-tail collagen and type IV collagenase were purchased from Sigma-Aldrich (Sigma-Aldrich, St. Louis, MO, USA). Briefly, the stock solution of DHA was dissolved in 95% ethanol at a concentration of 200 mM, and the working solution was prepared by adding the stock to the culture medium to achieve a final concentration of 25 μM. Epidermal growth factor was a product from Peprotech (Peprotech, Rocky Hill, NJ, USA). The primary antibodies applied in this study were anti-GRP78 (Cell Signaling Technology, Danvers, MA, USA), anti-ACC (Cell Signaling Technology, Danvers, MA, USA), anti-IRE (Santa Cruz Biotechnology, Santa Cruz, CA, USA), anti-ACOX1 (Abcam, Cambridge, UK), anti-p-IRE1α (Abcam, Cambridge, UK) and anti-β-actin (Beyotime, Shanghai, China).

### 2.2. Primary Mouse Hepatocytes Culture

Hepatocytes were prepared from male C57/6J mice referred to a modification of the two-step perfusion method as described previously [29]. The animals were anesthetized by intraperitoneal injection chloral hydrate (10 mL/kg, 4%). The abdominal cavity was opened, and the hepatic portal vein exposed. First, the liver was perfused with perfusion buffer 1 (calcium-free P1 medium) through a portal vein until the liver became pale in color; then perfusion buffer 2 supplemented with 0.035% type IV collagenase (P2 digestion medium) was used, keeping at a flow rate of 5 mL/min for about 6 min. The P1 and P2 medium should be warmed for 30 min in the water bath at 37 °C before use. After digestion, the hepatocytes were collected and washed with suspension medium, and then centrifuged at 50× *g* for 3 min at 4 °C twice. A cell count and cell viability assessment by trypan blue exclusion using a hemocytometer were performed. Freshly prepared hepatocytes were seeded at a final density of 1.5 × 10^6^ cells in collagen-coated 25 cm^2^ culture vessels, which were kept in tissue culture incubator set at 37 °C in a humidified atmosphere of 5% CO_2_ and 95% air. The cells were maintained in 10% FBS (Gibco^®^, South Melbourne, Victoria, Australia) DMEM/F12 medium (Gibco^®^, Shanghai, China) supplemented with 1 mL penicillin-streptomycin, then the medium was replaced with serum-free DMEM/F12 medium (supplement 100 units/mL penicillin, 100 μg/mL streptomycin, 10 μg/mL insulin, 0.1 μmol/L dexamethasone, 5 ng/mL epidermal growth factor) after 4 h.

### 2.3. Oil Red O Staining

The cells grown on glass coverslips were washed with phosphate buffered saline (PBS) three times and then fixed with 4% paraformaldehyde for 30 min at room temperature. The fixed cells were washed with PBS and stained with freshly diluted Oil Red O working solution (0.5% Oil Red O in isopropanol: H_2_O = 3:2) for 1 h, and counterstained with haematoxylin for 3 min. The primary mouse hepatocytes were observed using a microscope.

### 2.4. RNA Extraction and Real-Time PCR Assays

Total RNA was isolated from treated primary hepatocytes using Tripure Isolation Reagent (Roche, Mannheim, Germany) according to the manufacturer’s instructions. cDNA was synthesized with a Reverse Transcription Kit (TaKaRa, Otsu, Japan). Real-time PCR analysis was performed with SYBR Green in a thermal Cycler Dice Real Time System (TaKaRa, Otsu, Japan). The relative mRNA levels of target genes were assessed by using the 2^−ΔΔCt^ method. Each experiment was repeated three times. The sequences for the primers pairs were as follows (forward and reverse, respectively): *GADPH*: 5′-TGCTGTCCCTGTATGCCTCTG-3′ and 5′-TCTTTGATGTCACGCACGATTT-3′, *FAS*: 5′-GGCACTGACTGTCTGTTTTCCA-3′ and 5′-GTAAAAATGACACAGTCCAGACACTTC-3′, *ACC1*α: 5′-GTTTCAGAACGGCCACTACGA-3′ and 5′-CATTGTCACCAGGAGATTCTTTTTG-3′, *CPT1a*: 5′-TCTCTGGATGCGGTAGAAAAGG-3′ and 5′-CTCTATATCCCTGTTCCGATTCGT-3′, *Acox1*: 5′-GCCAATGCTGGTATCGAAGAA-3′ and 5′-AATCCCACTGCTGTGAGAATAGC-3′, *GRP78*: 5′-CAGGGCAACCGCATCAC-3′ and 5′-CAATCAGACGCTCCCCTTCA-3′, *XBP1*: 5′-AGTTAAGAACACGCTTGGGAT-3′ and 5′-AAGATGTTCTGGGGAGGTGAC-3′, *LXR*: 5′-AGGAGTGTGTGCTGTCAGAAGAAC-3′ and 5′-TCCTCTTCTTGCCGCTTCA-3′, *ChREBP*: 5′-CCCTCAGACACCCACATCTT-3′ and 5′-CAGAGCTCAGAAAGGGGTTG-3′, *SREBP1c*: , *CHOP*: 5′-GCATGAAGGAGAAGGAGCAG-3′ and 5′-CTTCCGGAGAGACAGACAGG-3′, *C/EBP1a*: 5′-CGCAAGAGCCGAGATAAAGC-3′ and 5′- CGGTCATTGTCACTGGTCAACT-3′.

### 2.5. Western Blot

Hepatocytes were lysed in RIPA buffer. Aliquots of 40 μg protein were loaded onto 8% sodium dodecyl sulfate-polyacrylamide gel electrophoresis (SDS-PAGE) gel, transferred to polyvinylidene difluoride (PVDF) membranes, and subsequently blocked with 5% nonfat milk for 1 h. The membranes were incubated with primary antibodies overnight, and then the secondary antibodies for 1 h. The protein bands were visualized with enhanced chemiluminiscence (ECL) detection system. The expression levels of protein were quantified with Fusion software.

### 2.6. Statistical Analysis

All experimental data were expressed as the mean ± SEM. Statistical differences were analyzed by one-way ANOVA, followed by Fisher’s least significant difference (LSD’s) multiple comparison test using SPSS 18.0 analysis software (SPSS, Chicago, IL, USA). Statistical significance was shown as * *p* < 0.05, ** *p* < 0.015, and *** *p* < 0.001.

## 3. Results

### 3.1. DHA Prevents Fructose-Induced Lipid Accumulation in Primary Mouse Hepatocytes

**Figure 1:**
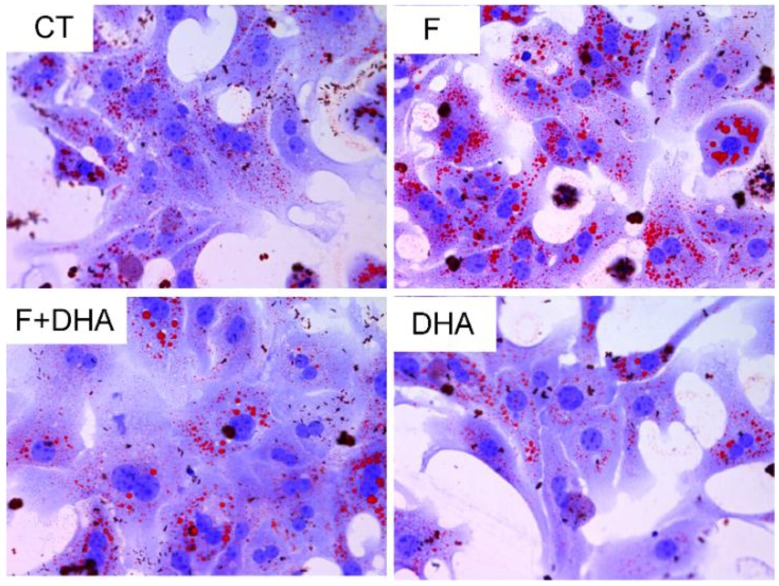
DHA ameliorates fructose induced TG accumulation in primary mouse hepatocytes Oil Red O staining. Original magnification: ×400; CT: control; F: fructose; F + DHA: fructose plus DHA.

To examine the effect of DHA on fructose treated primary mouse hepatocytes, cells were incubated for 24 h in DMEM/F12 medium containing 12.5 mM fructose (F), 12.5 mM fructose plus 25 μM DHA (F + DHA), or 25 μM DHA (DHA); control (CT) incubations only had vehicle. Then the cells were subjected to Oil Red O staining. After 24 h of incubation with fructose, the volume and numbers of lipid droplets were significantly increased indicating fructose treatment enhanced hepatic steatosis. In contrast, there was less triglyceride accumulation in DHA and F + DHA groups (Figure 1). Our data indicates that fructose treatment can cause TG accumulation and DHA may ameliorate this adverse effect.

### 3.2. DHA Attenuates Fructose-Induced Hepatic Steatosis Involving Changes in Expressions of Genes Related to Lipid Metabolism

**Figure 2:**
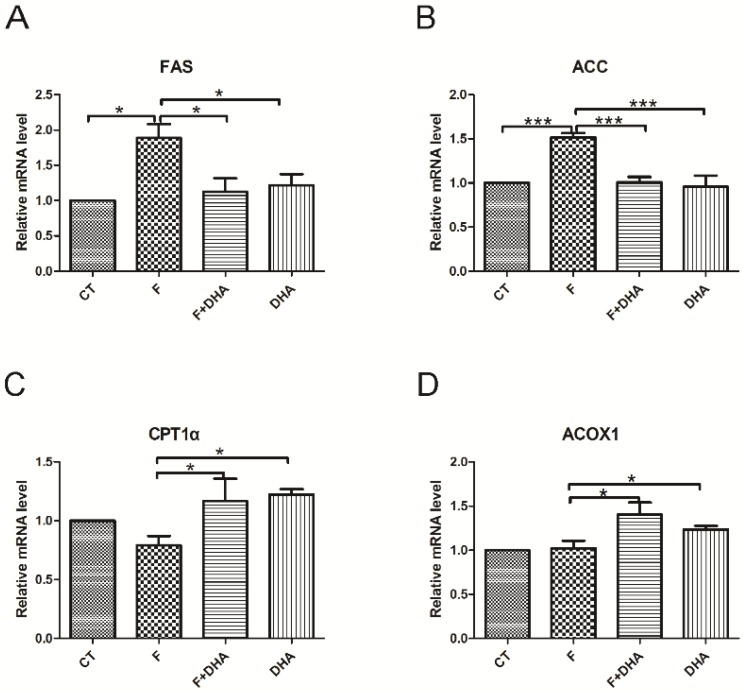
DHA regulates the expressions of genes involved in hepatic lipid metabolism. CT: control; F: fructose; F + DHA: fructose plus DHA. Expression values were normalized to control group. Data are expressed as mean ± SEM (*n* = 4). Data of the four groups were compared by ANOVA with LSD’s test (* *p* < 0.05).

To investigate the molecular basis for DHA preventing fructose-induced hepatic steatosis, we examined the expression of several genes involved in hepatic lipid metabolism using quantitative Real-Time PCR. The genes responsible for *de novo* lipogenesis, including *FAS* and *ACC*, were significantly up-regulated in the fructose treatment group (Figure 2A,B). Meanwhile, the other genes related to fatty acid oxidation, such as *CPT-1*α and *ACOX1* remained unchanged compared with control (Figure 2C,D). However, the DHA treatment group showed no significant increase of *FAS* and *ACC* expression compared with control (Figure 2A,B). In contrast, an upregulation of *CPT-1*α and *ACOX1* was observed in the DHA treatment groups (Figure 2C,D). These findings suggest that the ameliorating effect of DHA on fructose-induced hepatic steatosis was attributed to the increase in fatty acid oxidation and decrease in *de novo* lipogenesis.

### 3.3. ER Stress Pathways Mediates Fructose-Induced Lipid Accumulation

**Figure 3:**
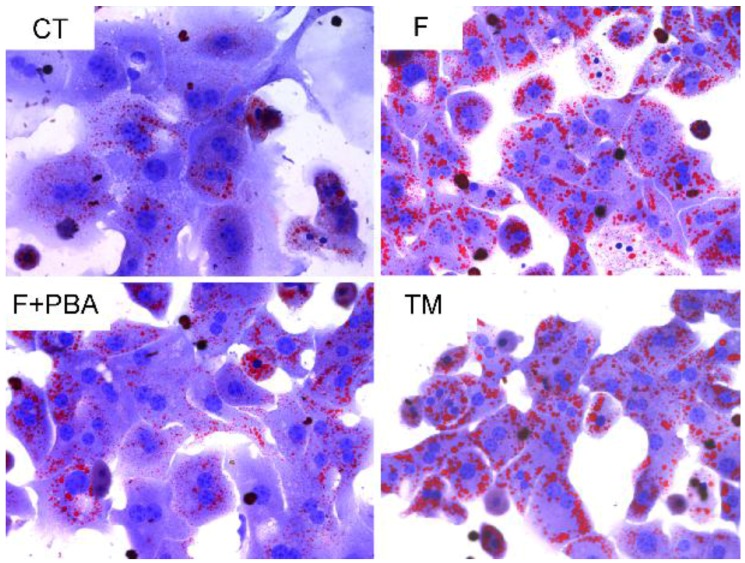
Effect of ER stress response on TG accumulation in primary mouse hepatocytes by treatment with ER stress inhibitor PBA or ER stress inducer TM. Oil Red O staining. Original magnification: ×400; CT: control; F: fructose; F + PBA: fructose plus PBA pretreatment; TM: tunicamycin.

**Figure 4:**
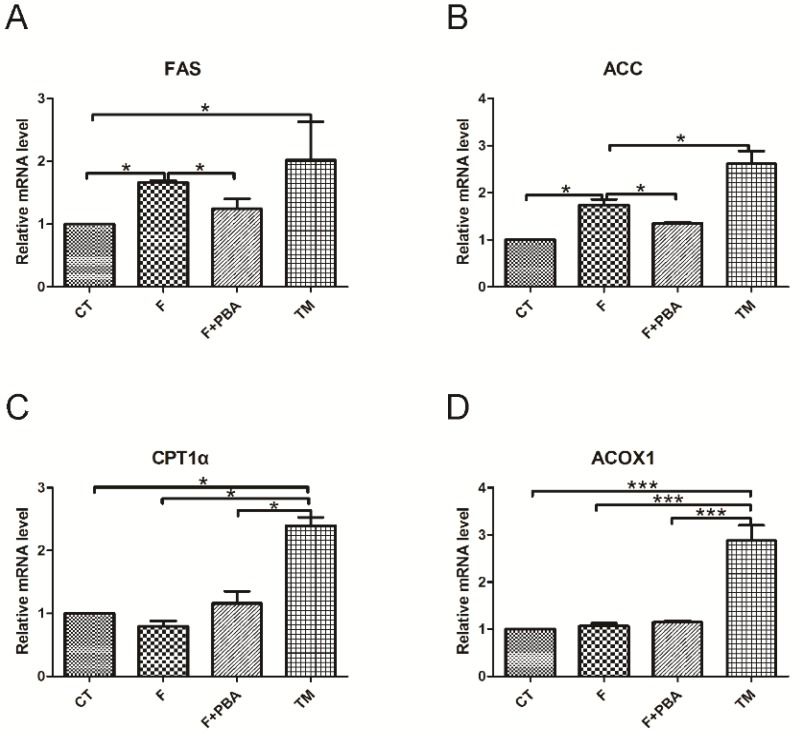
ER stress response mediated hepatic steatosis. CT: control; F: fructose; F + PBA: fructose plus PBA pretreatment; TM: tunicamycin. Expression values were normalized to control group. Data are expressed as mean ± SEM (*n* = 4). Data of the four groups were compared by ANOVA with LSD’s test (* *p* < 0.05, *** *p* < 0.001).

To test whether fructose triggered ER stress in hepatocytes, cells were treated with 2 μg/mL tunicamycin (TM), or pretreated with 2 mM 4-phenylbutyric acid for 1 h, and then incubated with 12.5 mM fructose (F + PBA) for 24 h respectively. Oil Red O staining showed increased TG accumulation in hepatocytes with treatment of fructose or the ER stress inducer TM. Interestingly, pretreatment with the ER stress inhibitor significantly decreased TG accumulation (Figure 3). Next, we investigated changes of mRNA levels of lipid homeostasis-related genes in response to ER stress. As illustrated in Figure 4A,B, compared with the control group, fructose and TM treatment increased the mRNA levels of *FAS* and *ACC*. However, pretreatment with the ER stress inhibitor PBA prevented these changes (Figure 4A,B). TM treatment significantly up-regulated *CPT-1*α and *ACOX1* expressions, but no changes were seen in the fructose treatment group and PBA pretreatment group (Figure 4C,D). Together, these findings suggest that fructose-induced hepatic steatosis is mediated by triggering the ER stress response.

### 3.4. ER Stress Response Is Involved in the Protective Effects of DHA against Fructose-Induced Hepatic Steatosis

**Figure 5:**
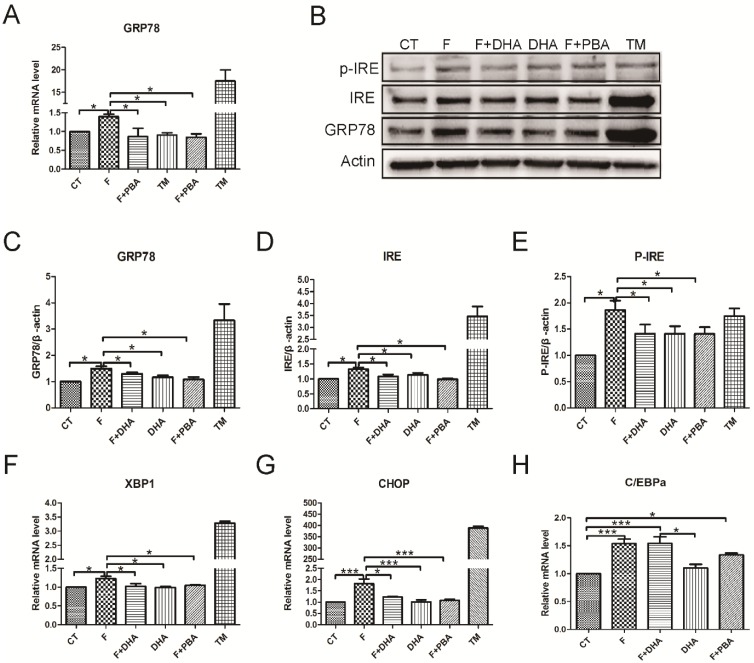
DHA alleviated fructose-induced ER stress response in primary mouse hepatocytes. CT: control; F: fructose; F + DHA: fructose plus DHA; F + PBA: fructose plus PBA pretreatment; TM: tunicamycin. Data are expressed as mean ± SEM (*n* = 4). Data of five groups were compared by ANOVA with LSD’s test (* *p* < 0.05, *** *p* < 0.001).

To further delineate the protective role of DHA in fructose-induced ER stress response and hepatic steatosis, we examined the changes of markers in the ER stress-activated unfolded protein response (UPR) pathways. Firstly, we examined the effects of DHA on chaperone expression using real-time PCR. The results indicated that DHA or PBA treatment significantly decreased fructose-induced upregulation of GRP78 in primary hepatocytes (Figure 5A). The reduction of GRP78 expression was further confirmed by Western blot (Figure 5B,C). Additionally, one of the markers of ER membrane protein IRE1α and its phosphorylated form p-IRE1α were drastically increased by fructose or TM treatment. However, both DHA and PBA prevented these changes (Figure 5B,D,E). The mRNA levels of X-box binding protein 1 (*XBP-1*) and C/EBP homologous protein (*CHOP*) were significantly elevated by fructose and TM treatment, whereas transcription factor *C/EBP*α mRNA level was not significantly up-regulated compared with DHA or PBA treatment (Figure 5F–H). These results suggest that DHA may alleviate the fructose-induced ER stress response in primary hepatocytes.

**Figure 6:**
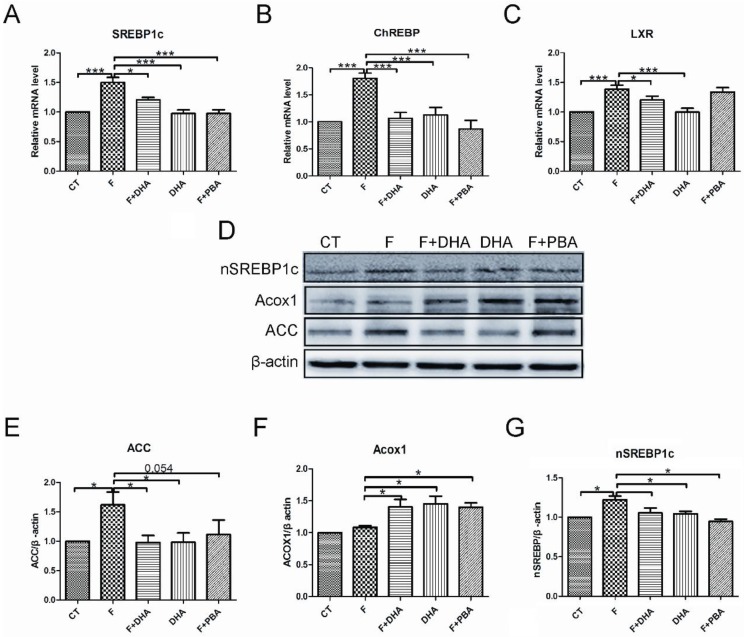
DHA selectively regulated gene expression related to lipid-homeostasis in primary mouse hepatocytes. CT: control; F: fructose; F + DHA: fructose plus DHA; F + PBA: fructose plus PBA pretreatment. Data are expressed as mean ± SEM (*n* = 4). Data of five groups were compared by ANOVA with LSD’s test (* *p* < 0.05, *** *p* < 0.001).

Next, we investigated whether DHA could affect the expression levels of hepatic lipid-homeostasis regulators using Western blot analysis or quantitative real-time PCR. First, we detected expression levels of some nuclear transcription factors which control hepatic *de novo* lipogenesis. As shown in Figure 6, the fructose-induced upregulation of liver X receptor (LXR) was suppressed by DHA treatment (Figure 6C). Both DHA and PBA treatment significantly decreased fructose-induced sterol-regulatory element-binding protein 1 (SREBP-1c) and carbohydrate responsive element binding protein (ChREBP) expression (Figure 6A,B,G). We next assessed the levels of ACC and ACOX1 which receive regulation from the above mentioned nuclear transcription factors. A decrease in ACC protein level was observed in cells treated with DHA or PBA (Figure 6E); however, ACOX1 levels were elevated in these groups (Figure 6F). Taken together, these findings indicate that DHA ameliorates fructose-induced hepatic steatosis by alleviating the ER stress response.

## 4. Discussion

Recent epidemiological and animals studies have strongly certified that the overconsumption of fructose is involved in the development of NAFLD [10,11,12]. Due to the relatively slow rate of progression from mild nonalcoholic fatty liver to more severe hepatitis or fibrosis and lack of approved pharmacotherapy for NAFLD [30], we have the opportunity to take some measurements to prevent the progression of NAFLD. Previous studies have shown that supplementation of DHA and EPA-rich fish oil has a beneficial effect on hepatic lipid metabolism [20,21]. Therefore, we speculate that DHA may have a therapeutic effect on fructose-induced hepatic steatosis.

In the present study, we demonstrated that fructose treatment of primary mouse hepatocytes induced an obvious hepatic steatosis observed by Oil Red O staining. This effect is attributable in part to its upregulation of lipid-related genes such as ACC and stearoyl-CoA desaturase (SCD) causing the ER stress response. The supplementation of DHA can prevent the adverse metabolic effects caused by fructose treatment. In addition, the fructose-provoked ER stress response was also inhibited by DHA. Taking together, these findings support the notion that DHA can ameliorate fructose-induced hepatic lipid accumulation through alleviating ER stress response.

NAFLD is characterized by increased triglyceride in the liver. The accumulation of hepatic lipid is attributed to increased *de novo* lipogenesis, increased fatty acid uptake, or reduced fatty acid oxidation [31]. In the current study, primary hepatocytes treated with fructose shown enhanced *de novo* lipogenesis, which is consistent with previous reports [10,11,12]. Some studies suggested that the lipogenic enzymes such as FAS and ACC were significantly up-regulated in fructose solution fed mice, which play important roles during hepatic *de novo* lipogenesis [17,32]. Moreover, fructose feeding increases the binding of LXR to the SREBP-1c promoter [33]. LXR is highly expressed in the liver, which induces SREBP-1c, FAS, ACC and SCD-1 transcription. Interestingly, ChREBP is also a direct target of LXR, which was found to be elevated in fructose treated hepatocytes. In the present study, DHA significantly reduced the expression levels of these key transcription factors and target enzymes in primary mouse hepatocytes, which is consistent with previous studies with fructose or fish oil feeding [20,34]. Another important finding was that DHA might ameliorate fructose-induced TG accumulation through increasing fatty acid oxidation. Hepatic *de novo* lipogenesis is considered to have an indirect effect on the increased levels of malonyl-CoA, which decreases the amount of fatty acid entering the mitochondria via restraining carnitine palmitoyltransferase 1 (CPT1) [35]. CPT1 is a rate-limiting enzyme of β-oxidation in the liver, which is necessary for long chain fatty acid entry into mitochondria for β-oxidation [36]. The current study found that the expression of CPT1 was significantly increased upon DHA treatment. Similarly, DHA elevates the expression of acyl-CoA oxidase 1 (ACOX1), an enzyme responsible for catalyzing peroxisomal β-oxidation of fatty acids. It seems that these results are due to the nuclear transcription factor activation by DHA as a PPARα ligand [37]. This findings further support the idea that DHA exerts its protective effects on fructose-induced hepatic steatosis through reducing key lipogenic enzymes expression and increasing fatty acid oxidation in hepatocytes.

Recently, ER stress response signaling has been tightly linked to hepatic lipid metabolism, insulin action, inflammation and apoptosis [26,27,38,39,40]. Previous studies showed that liver-specific IRE1α deletion and ATF6 knockout mice developed serious hepatic steatosis upon pharmacological ER stress [39,40]. Transcription factor XBP1 is a key regulator of the mammalian ER stress response as a downstream target of phosphorylated IRE1α. Moreover, it is implicated that XBP1 regulates hepatic lipogenesis unrelated to its role in the ER stress response [38]. Here, we found that fructose treatment caused the ER stress response in primary hepatocytes as evidenced by improved expression of ER membrane chaperone GRP78. One of the three ER-localized proteins IRE1α, and its activated form p-IRE1α were increased in fructose-treated hepatocytes. We found that DHA can alleviate the fructose-induced ER stress response as evidenced by down-regulation of the ER stress marker GRP78 and total IREα or p-IREα. This preventive effect of DHA was further proven by using ER stress inhibiter PBA and inducer TM. It has been demonstrated that TM induced pharmacological ER stress rapidly caused hepatic steatosis [41]; However, PBA alleviates ER stress in obese ob/ob mice and prevents hepatic TG accumulation [42,43]. This study confirms that DHA serves as an important dietary factor for NAFLD prevention and treatment. The protective effects are attributable, at least in part, to its roles of ER stress alleviation. All three unfolded protein response sensors, IRE1α, PERK and ATF6 are considered to play roles in lipid storage in the liver. In the current study, we found total IRE1α or p-IRE1α were upregulated by fructose. Activation of p-IRE1α promotes the splicing of *XBP-1* mRNA and subsequently produces a potent transcriptional activator. The current study was unable to evaluate the formation of XBP-1s, however, we indeed found that the XBP-1 mRNA level was upregulated upon fructose treatment. It is therefore likely that a sustained ER stress response exists, since the spliced form of XBP-1 can keep activating transcription by autoregulating its own transcription as far as IRE1α is activated [44]. The level changes of another downstream protein—CHOP, are similar to that of XBP-1. CHOP is a member of C/EBP family of transcriptional factors, and has been proposed to be a dominant-negative regulator of their function. Previous study suggests unresolved ER stress response will lead to suppression of *C/EBP*α partially through CHOP [27]. One limitation of this current investigation lies in the fact that we did not analyze the causative effects of DHA on ER stress response signaling pathways, and specifically what is the signal involved in DHA ameliorating fructose-induced hepatic steatosis. The results of this study do not explain the hierarchy of genetic regulation downstream of DHA treatment. Further studies on the current topic are therefore recommended.

In summary, the present study contributed to the existing knowledge that DHA prevents fructose-induced hepatic lipogenesis and accelerates fatty acid oxidation. The protective effect appears to be mediated through alleviating fructose-evoked ER stress response.

## 5. Conclusions

Increasing sugar consumption leads to higher fructose intakes, which is considered to be a risk factor for developing NAFLD. Therefore, life style changes and optimal dietary intervention beneficial to NAFLD are necessary. The present study confirms previous findings and contributes additional evidence that DHA ameliorates fructose-induced TG accumulation by preventing hepatic lipogenesis and enhancing fatty acid oxidation. More research is needed for better understanding the ER stress response signaling involved in these process. As a major ingredient in fish oil, DHA may have a therapeutic potential in the prevention and treatment of NAFLD.
